# Heterogeneity of DNA content in multiple synchronous hepatocellular carcinomas.

**DOI:** 10.1038/bjc.1997.387

**Published:** 1997

**Authors:** A. M. Hui, S. Kawasaki, H. Imamura, S. Miyagawa, K. Ishii, T. Katsuyama, M. Makuuchi

**Affiliations:** First Department of Surgery, Shinshu University School of Medicine, Matsumoto, Japan.

## Abstract

Heterogeneity of DNA content in multiple hepatocellular carcinomas (HCCs) was investigated by flow cytometry in 62 tumours from 26 patients who had undergone surgical treatment for multiple synchronous HCCs. Heterogeneity of DNA content was defined (a) when tumours had a different DNA ploidy pattern or (b) when the difference in the DNA index of the aneuploid clone was more than 0.1. A tumour with DNA aneuploidy was observed in 17 (66%) of the 26 patients. Heterogeneity of the DNA content was demonstrated in 12 (46%) out of 26 patients: in ten cases by definition (a) and in two cases by definition (b). Histological examination revealed that, of the 12 patients with a heterogeneous tumour DNA content, seven (58%) had a heterogeneous and the remaining five (42%) had a homogeneous type and grade of differentiation among the tumours, showing the absence of a relationship between histological heterogeneity and DNA content. The present results suggest the clinical relevance of DNA content analysis for identifying the clonal origin of multiple HCCs.


British Journal of Cancer (1997) 76(3), 335–339
© 1997 Cancer Research Campaign

### Heterogeneity of DNA content in multiple synchronous hepatocellular carcinomas

**A-M Hui[1], S Kawasaki[1], H Imamura[1], S Miyagawa[1], K Ishii[2], T Katsuyama[2] and M Makuuchi[3]**

[1]First Department of Surgery, Shinshu University School of Medicine; [2]Central Clinical Laboratories, Shinshu University Hospital, Matsumoto, Japan; [3]Second Department of Surgery, University of Tokyo, Faculty of Medicine, Tokyo, Japan

**Summary** Heterogeneity of DNA content in multiple hepatocellular carcinomas (HCCs) was investigated by flow cytometry in 62 tumours from 26 patients who had undergone surgical treatment for multiple synchronous HCCs. Heterogeneity of DNA content was defined (a) when tumours had a different DNA ploidy pattern or (b) when the difference in the DNA index of the aneuploid clone was more than 0.1. A tumour with DNA aneuploidy was observed in 17 (66%) of the 26 patients. Heterogeneity of the DNA content was demonstrated in 12 (46%) out of 26 patients: in ten cases by definition (a) and in two cases by definition (b). Histological examination revealed that, of the 12 patients with a heterogeneous tumour DNA content, seven (58%) had a heterogeneous and the remaining five (42%) had a homogeneous type and grade of differentiation among the tumours, showing the absence of a relationship between histological heterogeneity and DNA content. The present results suggest the clinical relevance of DNA content analysis for identifying the clonal origin of multiple HCCs.

**Keywords:** hepatocellular carcinoma; DNA content; heterogeneity; flow cytometry

Hepatocellular carcinoma (HCC) is the most common malignancy in Africa and Asia and is associated with a poor prognosis. Surgical resection is the first choice of treatment. However, the frequent development of multiple tumours in patients with chronic viral hepatitis and/or cirrhosis hinders curability. Thus, it is thought important to understand the biological behaviour and clonal origin of different tumours in cases of multiple HCC for evaluation of clinical stage, prediction of post-operative outcome and choice of suitable therapeutic treatments.

Flow cytometric analysis of cellular DNA content has become an increasingly important clinical tool for the evaluation of tumour biological behaviour. Quantitative DNA analysis reflects the total chromosomal content of tumour cells. In terms of the cell nuclear DNA content of HCC, it has been reported (Fujimoto et al, 1991; Chiu et al, 1992) that an aneuploid DNA pattern indicates poor prognosis. With regard to the heterogeneity of the DNA ploidy pattern, HCC has been reported to be mostly homogeneous within the same tumour (Kuo et al, 1987; Nagasue et al, 1993; Ng et al, 1994). However, there have been few studies on the heterogeneity of DNA content among multiple synchronous HCCs. In the present study, we investigated tumour DNA heterogeneity in patients with multiple synchronous HCCs.

### MATERIALS AND METHODS

Sixty-two tumours from 26 patients who had undergone surgical treatment for multiple HCCs at the First Department of Surgery, Shinshu University Hospital, between October 1989 and August 1994, were studied. Two patients each had four tumours, six patients had three tumours and the remaining 18 patients had two tumours each. There were 17 men and nine women with a mean age of 64.5 ± 6.3 (s.d.) years (range, 54–82 years). In all patients, histological examination of the non-cancerous tissue demonstrated chronic hepatitis, precirrhosis or cirrhosis. Three patients were positive for hepatitis B virus, 22 patients for hepatitis C virus and one patient for both viruses. The tumour size ranged from 0.6 to 6.0 cm with an average of 2.4 ± 1.1 cm (mean ± s.d.). These 62 tumours in the present study were considered to be of potentially multifocal origin as they did not meet the following criteria of intrahepatic metastasis: multiple HCCs were diagnosed as metastatic in origin from a single tumour (a) when they were components of portal vein tumour thrombi, i.e. tumours apparently growing in contiguity with portal vein tumour thrombi or (b) when they were distributed as multiple small-satellite nodules surrounding a large main tumour (Kanai et al, 1987; Oda et al, 1992; Tsuda et al, 1992).

A cell nucleus suspension was obtained from a paraffin-embedded block of a surgically resected specimen, following the methods of Hedley with some modifications (Hedley et al, 1983; Hui et al, 1994). Briefly, the tumour and non-tumour parts were obtained from resected specimens. Three 50-μm-thick sections were cut, in addition to a 5-μm-thick section before and after each series of 50-μm-thick sections. The 5-μm-thick section was stained with haematoxylin and eosin in order to evaluate the pathological features and to assess the tumour area in the section, then accordingly, the tumour was trimmed from the 50-μm-thick section. The tissue was dewaxed, rehydrated and then digested with pepsin. The isolated nuclei were stained according to the method described previously (Vindeløv et al, 1983). Cell nuclear DNA content was analysed using a FACScan (Becton Dickinson, Mountain View, CA, USA) with Cellfit doublet discriminator software. The histogram was derived from up to 15 000 cells. The instrument was calibrated with chicken erythrocyte nuclei. As external controls, sections from non-tumour liver within the same surgical specimen were processed

*Received 5 July 1996*
*Revised 2 January 1997*
*Accepted 13 January 1997*

*Correspondence to:* Seiji Kawasaki, First Department of Surgery, Shinshu University School of Medicine, 3-1-1 Asahi, Matsumoto 390, Japan

**Figure 1** DNA ploidy pattern. Three patterns of nuclear DNA ploidy status were classified. Pattern I, diploidy (DNA index from 0.9 to 1.1) (**A**). Pattern II, aneuploidy with one $G_0/G_1$ peak, either hypoploidy (DNA index < 0.9) (**B**) or hyperploidy (DNA index > 1.1) (**C**). Pattern III, aneuploidy with more than one $G_0/G_1$ peak (**D**)

simultaneously with the tumour sample, and the DNA index (DI) was calculated according to the standard method (Hiddemann et al, 1984). Tumours with a DI of 0.9–1.1 were recorded as diploid, and those with a DI of more than 1.1 or less than 0.9 as aneuploid. The DNA ploidy status of the tumour was classified into three patterns (Chiu et al, 1992): pattern I, tumours with a diploid DNA distribution; pattern II, tumours with an aneuploid DNA content and containing only one prominent $G_0/G_1$ peak; pattern III, tumours with an aneuploid DNA content and containing > 1 $G_0/G_1$ peaks (Figure 1). Heterogeneity of DNA content among multiple tumours was recognized when tumours had different DNA ploidy patterns or when the difference in the DI of an aneuploid clone exceeded 0.1, even though the tumours belonged to the same pattern II or pattern III. The coefficient of variation (CV) of the diploid $G_0/G_1$ peak ranged from 3.5% to 8.7% (mean ± s.d., 5.53 ± 1.35%).

### RESULTS

The results of histological and flow cytometric studies of the 62 tumours from 26 patients with multiple synchronous HCCs are summarized in Table 1. The tumours had a trabecular pattern in 53 cases (85.5%), a compact pattern in six (9.7%), a pseudoglandular pattern in two (3.2%) and a solid pattern in one (1.6%). Among the 26 patients, six (23.1%) had heterogeneous and 20 (76.9%) had homogeneous histological type among the multiple tumours. Regarding the grade of tumour differentiation, 20 tumours (32.3%) were well differentiated, 33 (53.2%) were moderately differentiated and nine (14.5%) were poorly differentiated. The histological type and the grades of differentiation among the multiple tumours were identical in 9 of the 26 cases and different in the remaining 17. A tumour with aneuploid DNA was observed in 17 (65.4%) of the 26 patients. Among the 62 tumours, 35 (56.4%) were diploid (pattern I) and 27 (43.5%) were aneuploid, of which 14 tumours belonged to pattern II and the remaining 13 to pattern III. DNA heterogeneity among multiple tumours was observed in 12 out of 26 patients (46.2%, cases 15–26), among which the DNA content difference represented the diploid–aneuploid type in ten (cases 17–26). In cases 15 and 16, the difference in DI of the aneuploid clone was more than 0.1, although both of the tumours were aneuploid (pattern III). Of the 12 patients with tumours showing DNA heterogeneity, seven (58.3%) had the heterogeneous (cases 15, 18, 19, 20, 23, 24 and 26) and the remaining five (41.7%) had the homogeneous histological type and grade of tumour differentiation.

### DISCUSSION

Quantitative DNA analysis reflects the total chromosomal content of tumour cells and it can now be conducted rapidly and reliably using flow cytometry. Flow cytometric DNA analysis has become a valuable adjunct to the clinical and histopathological assessment of cancers.

© Cancer Research Campaign 1997

**Table 1** Histopathological features and DNA content in multiple hepatocellular carcinoma

| Case | Tumour | Histological type | Differentiation | DNA analysis | | | Histological type heterogeneity | Differentiation heterogeneity | DNA content heterogeneity |
|---|---|---|---|---|---|---|---|---|---|
| | | | | Pattern | CV (%) | DI | | | |
| 1 | T1 | Trabecular | Well | I[1] | 6.8 | 1.00 | | | |
| | T2 | Trabecular | Well | I | 3.6 | 1.00 | No | No | No |
| 2 | T1 | Trabecular | Well | I | 6.1 | 1.02 | | | |
| | T2 | Trabecular | Well | I | 5.4 | 1.02 | No | No | No |
| 3 | T1 | Trabecular | Well | I | 4.2 | 1.05 | | | |
| | T2 | Trabecular | Moderate | I | 4.6 | 1.02 | | | |
| | T3 | Trabecular | Well | I | 3.5 | 1.02 | No | Yes | No |
| 4 | T1 | Trabecular | Well | I | 5.8 | 1.08 | | | |
| | T2 | Trabecular | Moderate | I | 7.3 | 1.03 | No | Yes | No |
| 5 | T1 | Trabecular | Moderate | I | 7.6 | 1.06 | | | |
| | T2 | Trabecular | Moderate | I | 6.1 | 1.03 | | | |
| | T3 | Trabecular | Poor | I | 5.4 | 1.03 | No | Yes | No |
| 6 | T1 | Trabecular | Moderate | I | 4.0 | 0.98 | | | |
| | T2 | Trabecular | Moderate | I | 4.5 | 0.95 | No | No | No |
| 7 | T1 | Trabecular | Moderate | I | 6.7 | 1.01 | | | |
| | T2 | Solid | Poor | I | 5.4 | 1.01 | | | |
| | T3 | Trabecular | Well | I | 7.5 | 1.02 | | | |
| | T4 | Trabecular | Well | I | 7.5 | 0.96 | Yes | Yes | No |
| 8 | T1 | Trabecular | Moderate | I | 6.4 | 1.06 | | | |
| | T2 | Trabecular | Moderate | I | 4.2 | 1.10 | | | |
| | T3 | Trabecular | Moderate | I | 5.8 | 1.04 | No | No | No |
| 9 | T1 | Trabecular | Moderate | I | 4.2 | 1.08 | | | |
| | T2 | Trabecular | Well | I | 4.8 | 0.94 | No | Yes | No |
| 10 | T1 | Trabecular | Well | II | 3.9 | 1.22 | | | |
| | T2 | Pseudoglandular | Moderate | II | 5.9 | 1.31 | Yes | Yes | No |
| 11 | T1 | Compact | Poor | II | 6.5 | 1.14 | | | |
| | T2 | Trabecular | Poor | II | 4.5 | 1.16 | Yes | No | No |
| 12 | T1 | Trabecular | Well | II | 4.7 | 1.19 | | | |
| | T2 | Trabecular | Moderate | II | 4.3 | 1.15 | No | Yes | No |
| 13 | T1 | Compact | Moderate | II | 4.6 | 1.18 | | | |
| | T2 | Trabecular | Moderate | II | 4.7 | 1.18 | Yes | No | No |
| 14 | T1 | Trabecular | Moderate | III | 5.3 | 1.29 | | | |
| | T2 | Trabecular | Moderate | III | 4.6 | 1.19 | No | No | No |
| 15 | T1 | Trabecular | Moderate | III | 5.2 | 1.22 | | | |
| | T2 | Trabecular | Well | III | 6.4 | 1.35 | No | Yes | Yes |
| 16 | T1 | Trabecular | Moderate | III | 8.7 | 1.24 | | | |
| | T2 | Trabecular | Moderate | III | 4.2 | 1.43 | No | No | Yes |
| 17 | T1 | Compact | Poor | I | 3.5 | 0.97 | | | |
| | T2 | Compact | Poor | II | 8.4 | 1.11 | No | No | Yes |
| 18 | T1 | Trabecular | Moderate | I | 5.8 | 0.95 | | | |
| | T2 | Trabecular | Well | II | 7.3 | 0.82 | No | Yes | Yes |
| 19 | T1 | Trabecular | Well | I | 4.9 | 0.96 | | | |
| | T2 | Trabecular | Poor | I | 4.7 | 0.93 | | | |
| | T3 | Trabecular | Well | II | 7.0 | 0.80 | No | Yes | Yes |
| 20 | T1 | Trabecular | Well | I | 4.6 | 1.06 | | | |
| | T2 | Trabecular | Well | II | 4.9 | 1.13 | | | |
| | T3 | Trabecular | Well | II | 4.3 | 1.22 | No | Yes | Yes |
| 21 | T1 | Trabecular | Moderate | I | 6.1 | 0.94 | | | |
| | T2 | Trabecular | Moderate | III | 7.3 | 0.79 | No | No | Yes |
| 22 | T1 | Trabecular | Moderate | I | 8.1 | 0.94 | | | |
| | T2 | Trabecular | Moderate | III | 5.9 | 2.16 | No | No | Yes |
| 23 | T1 | Trabecular | Moderate | I | 5.6 | 0.91 | | | |
| | T2 | Pseudoglandular | Moderate | III | 6.9 | 1.36 | Yes | No | Yes |
| 24 | T1 | Trabecular | Moderate | I | 7.5 | 0.99 | | | |
| | T2 | Trabecular | Well | III | 6.0 | 1.46 | No | Yes | Yes |
| 25 | T1 | Trabecular | Moderate | I | 5.0 | 1.01 | | | |
| | T2 | Trabecular | Moderate | I | 7.0 | 1.05 | | | |
| | T3 | Trabecular | Moderate | III | 4.6 | 1.25 | No | No | Yes |
| 26 | T1 | Trabecular | Moderate | I | 6.5 | 0.93 | | | |
| | T2 | Trabecular | Moderate | II | 8.1 | 0.82 | | | |
| | T3 | Compact | Poor | III | 4.8 | 1.77 | | | |
| | T4 | Compact | Poor | III | 6.3 | 1.93 | Yes | Yes | Yes |

[1]DNA ploidy pattern; CV, coefficient of variation; DI, DNA index; T, tumour.

© *Cancer Research Campaign 1997*

Although intratumoral heterogeneity of DNA content has been observed in carcinomas of various organs (Vindeløv et al, 1980; Quirke et al, 1983; Ljungberg et al, 1985; Sasaki et al, 1988; Sasaki et al, 1991), HCC is generally thought to be homogeneous within the tumour in terms of DNA content (Kuo et al, 1987; Nagasue et al, 1993; Ng et al, 1994), indicating that single sampling is sufficient for deciding the DNA ploidy pattern in patients with solitary HCC. In the present study, three 50-μm sections were prepared for flow cytometry procedures. Considering that materials are usually taken by one-site sampling in studies using fresh tissue, the results obtained from paraffin-embedded blocks, as in the present study, are considered more representative of the whole tumour than those from fresh materials, at least in terms of DNA content.

It has been shown that DNA content analysis is an important predictor of prognosis, which is better in diploid than in aneuploid tumours (Fujimoto et al, 1991; Chiu et al, 1992), although some authors have suggested that the advanced tumour stage of large lesions may negate the influence of DNA ploidy status on patient outcome (McEntee et al, 1992). These studies were limited to analysis of a single tumour nodule in patients with HCC. However, multiple HCCs occur in some cases. Heterogeneity of DNA content between different nodules, if present, may influence the DNA content status for predicting prognosis. In the present investigation, it was demonstrated that heterogeneity of DNA content among different tumour nodules occurred in 46% of synchronous multiple HCCs, indicating that analysis of the DNA content of every tumour is necessary for determing the prognosis in this disease.

Heterogeneity of DNA content in synchronous multiple HCCs has been demonstrated previously by Feulgen DNA analysis (Kuo et al, 1987) and flow cytometric DNA analysis (Nagasue et al, 1992). Unfortunately, however, the number of cases investigated was limited (14 cases in each study). The incidence of heterogeneity was 29% in the former study and 36% in the latter, which was lower than in our study (46%). There are two possible reasons for the discrepancy between the previous studies and ours. First, the previous studies included only patients with two tumour nodules, whereas in our study eight patients had more than two nodules. Second, the previous studies investigated heterogeneity simply by classifying tumours as either diploid or aneuploid (Nagasue et al, 1992), or by roughly comparing their DNA ploidy profiles (Kuo et al, 1987). By contrast, in the present study, heterogeneity of DNA content was considered not only when a different DNA ploidy pattern was observed, but also when a different DI (difference > 0.1) was demonstrated among the tumours with an aneuploid pattern.

Heterogeneity by histological criteria (histological type and grade of tumour differentiation) was observed in 16 (62%) of the 26 patients with synchronous multiple HCCs, and there seemed to be no relationship between heterogeneity of histological appearance and DNA content (Table 1).

Genetic and/or chromosomal analyses have demonstrated that solitary HCCs are of monoclonal origin (Esumi et al, 1986; Aihara et al, 1996). On the other hand, the clonal origin of multiple HCCs has been classified conventionally as multifocal or metastatic, i.e. polyclonal or monoclonal, based mainly on macro- and microscopic features. However, it has usually been difficult to determine clonal origin in clinical practice. Integrated HBV DNA has been used as a marker of HCC clonality, but this is applicable only to a portion of patients who have hepatitis B virus infection (Esumi et al, 1986; Tsuda et al, 1988; Sakamoto et al, 1989; Hsu et al, 1991). In our present series, only four patients harboured hepatitis B virus. Recently, examinations of chromosal allele loss patterns (Tsuda et al, 1992) and patterns of p53 gene mutation (Oda et al, 1992) have been reported to be useful for diagnosis of multifocal HCC. However, these methods are not totally ideal because p53 gene mutation and chromosal allele loss were detected in only 60–70% of multiple HCCs. These findings showed that judgement of the origin of multiple HCCs should be made on a comprehensive basis, taking into acount both morphology and other criteria such as DNA content.

Assuming that single HCCs are monoclonal and that their DNA content remains homogeneous during tumour growth, the difference in DNA content among multiple tumours strongly indicates their different clonal origin. However, analysis of DNA content must be considered limited, as it only reflects the chromosomal content of tumour cells, and a cell with a balanced gain or loss of chromosomes, or with structural rearrangements, will not be detected. Also, it can not give conclusive information on the clonal origin in cases showing a homogeneous DNA pattern, as tumours of different clonal origin may well present the same DNA ploidy pattern. In our series of multiple HCCs, multifocal origin was identified in 12 (46%) of the 26 patients by quantitative analysis of DNA content. In the remaining 14 (56%) cases in which the DNA content was homogeneous among the tumours, their clonal origin still remained undetermined. Comparing the various genetic methods for analysis of multifocal HCC, quantitative analysis of DNA content seems less specific, but it is simple and technically convenient. In order to estimate the value of quantitative DNA content analysis for deciding the clonal origin of multiple HCCs, further investigations such as comparative studies of genetic methods in a single series of patients with synchronous multiple HCCs will be necessary.

### REFERENCES

Aihara T, Noguchi S, Sasaki Y, Nakano H, Monden M and Imaoka S (1996) Clonal analysis of precancerous lesion of hepatocellular carcinoma. *Gastroenterology* **111**: 455–461

Chiu JH, Kao HL, Wu LH, Chang HM and Lui WY (1992) Prediction of relapse or survival after resection in human hepatomas by DNA flow cytometry. *J Clin Invest* **89**: 539–545

Esumi M, Aritaka T, Arii M, Suzuki K, Tanikawa K, Mizuo H, Mima T and Shikata T (1986) Clonal origin of human hepatoma determined by integration of hepatitis B virus DNA. *Cancer Res* **46**: 5767–5771

Fujimoto J, Okamoto E, Yamanaka N, Toyosaka A and Mitsunobu M (1991) Flow cytometric DNA analysis of hepatocellular carcinoma. *Cancer* **67**: 939–944

Hedley DW, Friedlander ML, Taylor IW, Rugg CA and Musgrove EA (1983) Method for analysis of cellular DNA content of paraffin-embedded pathological material using flow cytometry. *J Histochem Cytochem* **31**: 1333–1335

Hiddemann W, Schumann J, Andreef M, Barlogie B, Herman CJ, Leif RC, Mayall BH, Murphy RF and Sandberg AA (1984) Convention on nomenclature for DNA cytometry. Committee on Nomenclature, Society for Analytical Cytology. *Cancer Genet Cytogent* **13**: 181–183

Hsu HC, Chiou TJ, Chen JY, Lee CS, Lee PH and Peng SY (1991) Clonality and clonal evolution of hepatocellular carcinoma with multiple nodules. *Hepatology* **13**: 923–928

Hui AM, Itaboshi M, Kato H, Tachimori Y, Watanabe H and Hirota T (1994) Flow cytometric DNA analysis of submucosal carcinoma of the esophagus. *Jpn J Clin Oncol* **24**: 26–31

Kanai T, Hirohashi S, Upton MP, Noguchi M, Kishi K, Makuuchi M, Yamasaki S, Hasegawa H, Takayasu K, Moriyama N and Shimosato Y (1987) Pathology of small hepatocellular carcinoma: a proposal for a new gross classification. *Cancer* **60**: 810–819

Kuo SH, Shen JC, Chen DS, Sung JL, Lin CC and Hsu HC (1987) DNA clonal heterogeneity of hepatocellular carcinoma demonstrated by Feulgen-DNA analysis. *Liver* **7**: 350–363

© Cancer Research Campaign 1997

Ljungberg B, Stenling R and Roos G (1985) DNA content in renal cell carcinoma with reference to tumour heterogeneity. *Cancer* **56**: 503–508

McEntee GP, Batts KA, Katzmann JA, Ilstrup DM and Nagorney DM (1992) Relationship of nuclear DNA content to clinical and pathologic findings in patients with primary hepatic malignancy. *Surgery* **111**: 376–379

Nagasue N, Kohno H, Chang YC, Yamanoi A, Kimoto T, Takemoto Y and Nakamura T (1992) DNA ploidy pattern in synchronous hepatocellular carcinomas *J Hepatol* **16**: 208–214

Nagasue N, Kohno H, Hayashi T, Yamanoi A, Uchida M, Takemoto Y, Makino Y, Ono T, Hayashi J and Nakamura T (1993) Lack of intratumoural heterogeneity in DNA ploidy pattern of hepatocellular carcinoma. *Gastroenterology* **105**: 1449–1454

Ng IOL, Lai ECS, Ho JCW, Cheung LKN, Ng MMT and So MKP (1994) Flow cytometric analysis of DNA ploidy in hepatocellular carcinoma. *Am J Clin Pathol* **102**: 80–86

Oda T, Tsuda H, Scarpa A, Sakamoto M and Hirohashi S (1992) Mutation pattern of the *p53* gene as a diagnostic marker for multiple hepatocellular carcinomas. *Cancer Res* **52**: 3674–3678

Sakamoto M, Hirohashi S, Tsuda H, Shimosato Y, Makuuchi M and Hosoda Y (1989) Multicentric development of hepatocellular carcinoma revealed by analysis of hepatitis B virus integration pattern. *Am J Surg Pathol* **13**: 1064–1066

Sasaki K, Hashimoto T, Kawachino K and Takahashi M (1988) Intratumoural origional differences in DNA ploidy of gastrointestinal carcinomas. *Cancer* **62**: 2569–2575

Sasaki K, Murakami T, Murakami T and Nakamura M (1991) Intratumoural heterogeneity in DNA ploidy of esophageal cell carcinomas. *Cancer* **68**: 2403–2406

Tsuda H, Hirohashi S, Shimosato Y, Terata M and Hasegawa H (1988) Clonal origin of atypical adenomatous hyperplasia of liver and clonal identity with hepatocellular carcinoma. *Gastroenterology* **95**: 1664–1666

Tsuda H, Oda T, Sakamoto M and Hirohashi S (1992) Different pattern of chromosomal allele loss in multiple hepatocellular carcinomas as evidence of their multifocal origin. *Cancer Res* **52**: 1504–1509

Quirke P, Dyson JED, Dixon MF, Bird CC and Joslin CAF (1985) Heterogeneity of colorectal adenocarcinomas evaluated by flow cytometry and histopathology. *Br J Cancer* **51**: 99–106

Vindeløv LL, Hansen HH, Christensen IJ, Spang-Thomsen M, Hirsch FR, Hansen M and Nissen NI (1980) Clonal heterogeneity of small-cell anaplastic carcinoma of the lung demonstrated by flow-cytometric DNA analysis. *Cancer Res* **40**: 4295–4300

Vindeløv LL, Christensen IJ and Nissen NI (1983) A detergent-trypsin method for the preparation of nuclei for flow cytometric DNA analysis. *Cytometry* **3**: 323–327

© Cancer Research Campaign 1997


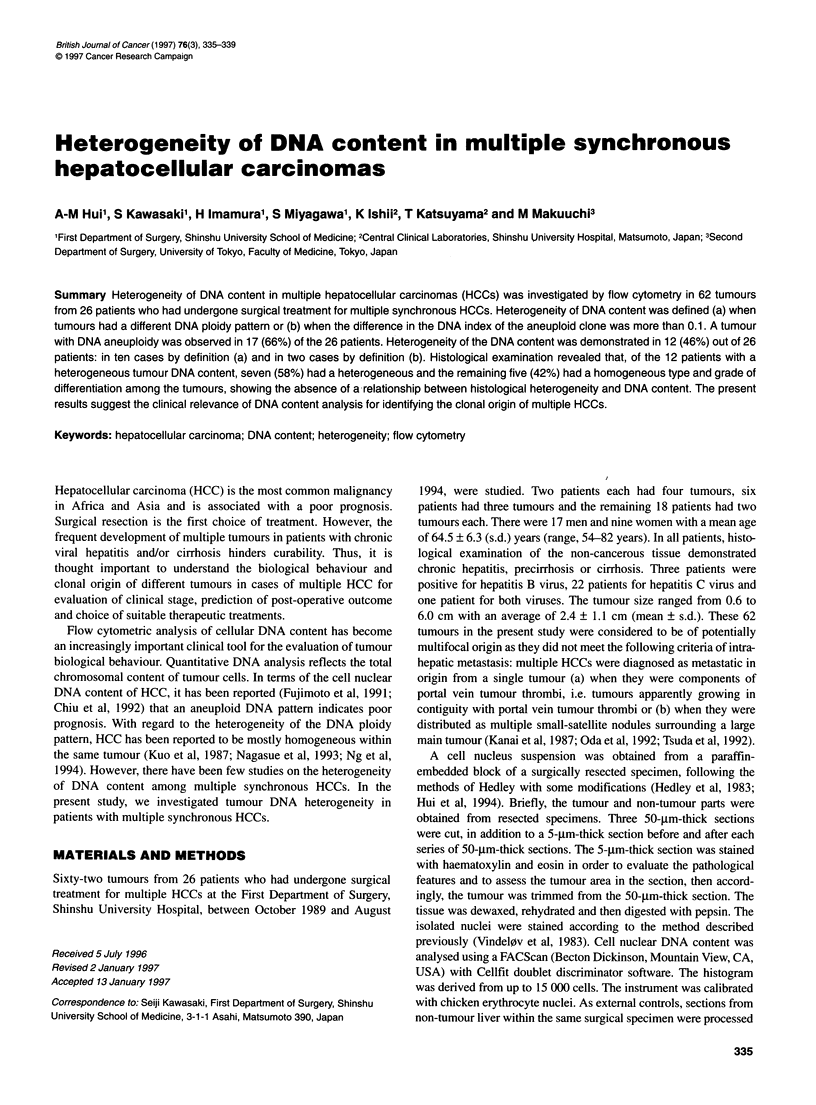

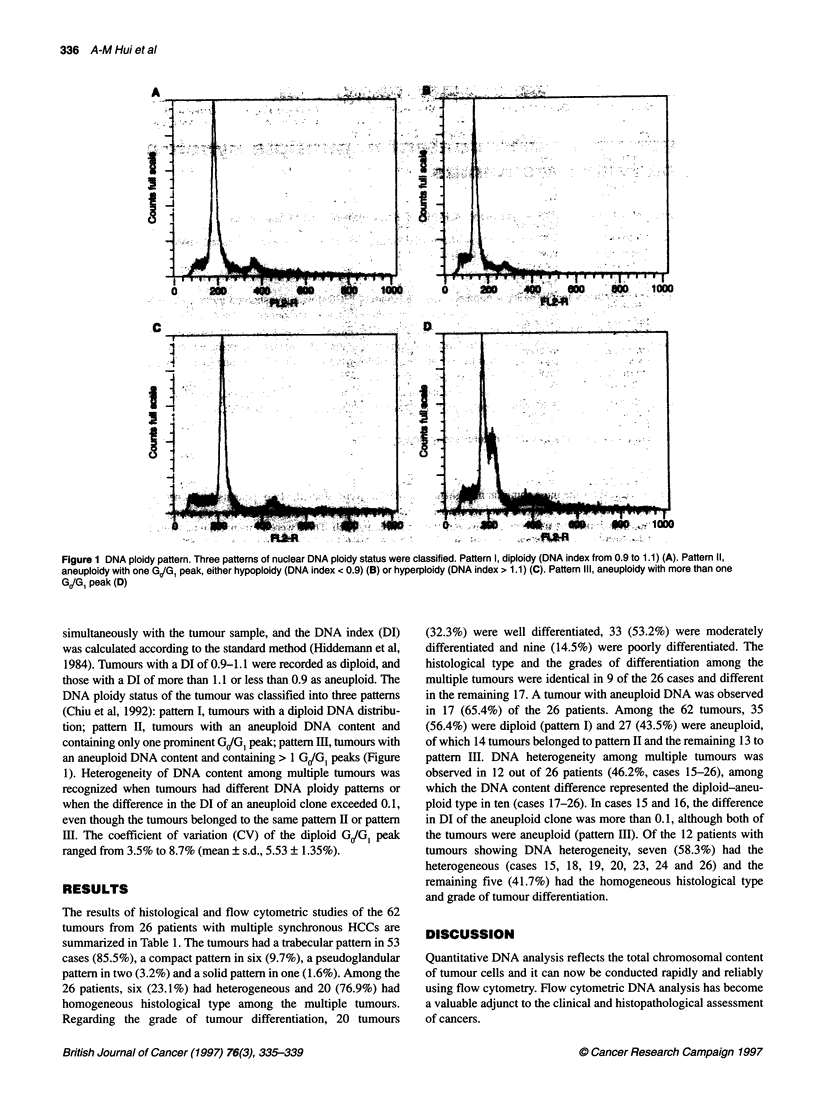

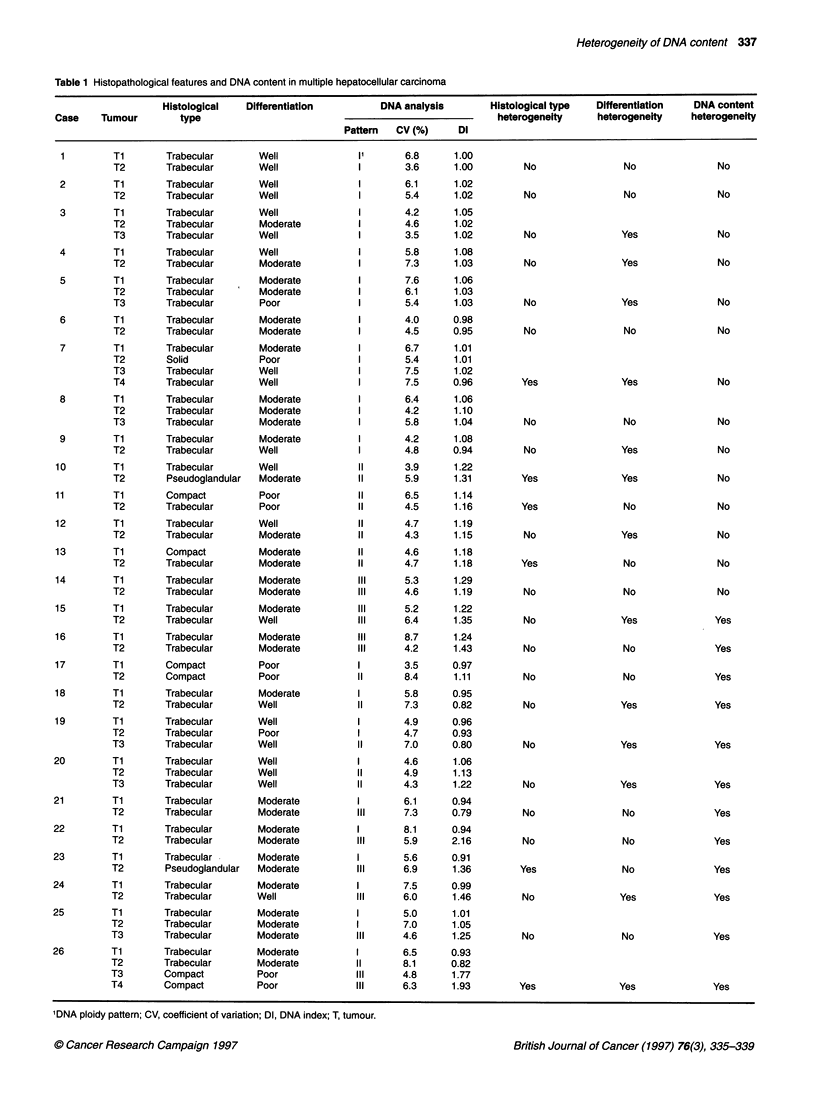

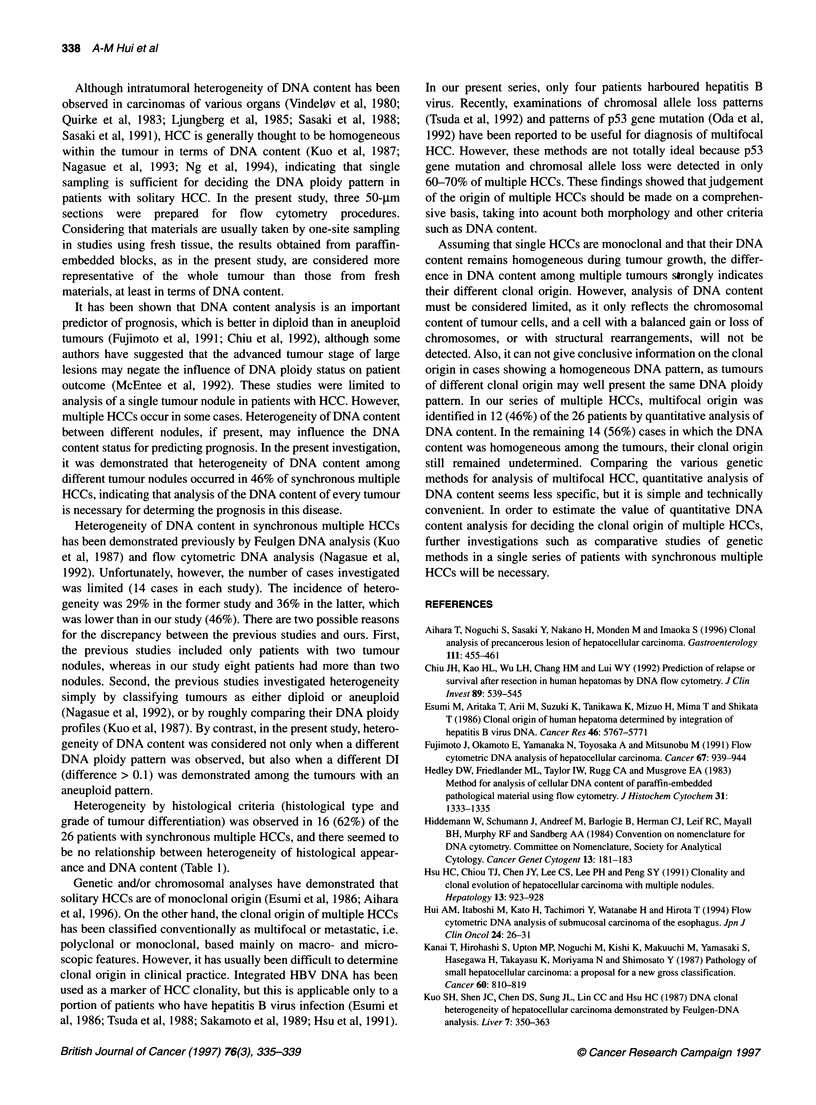

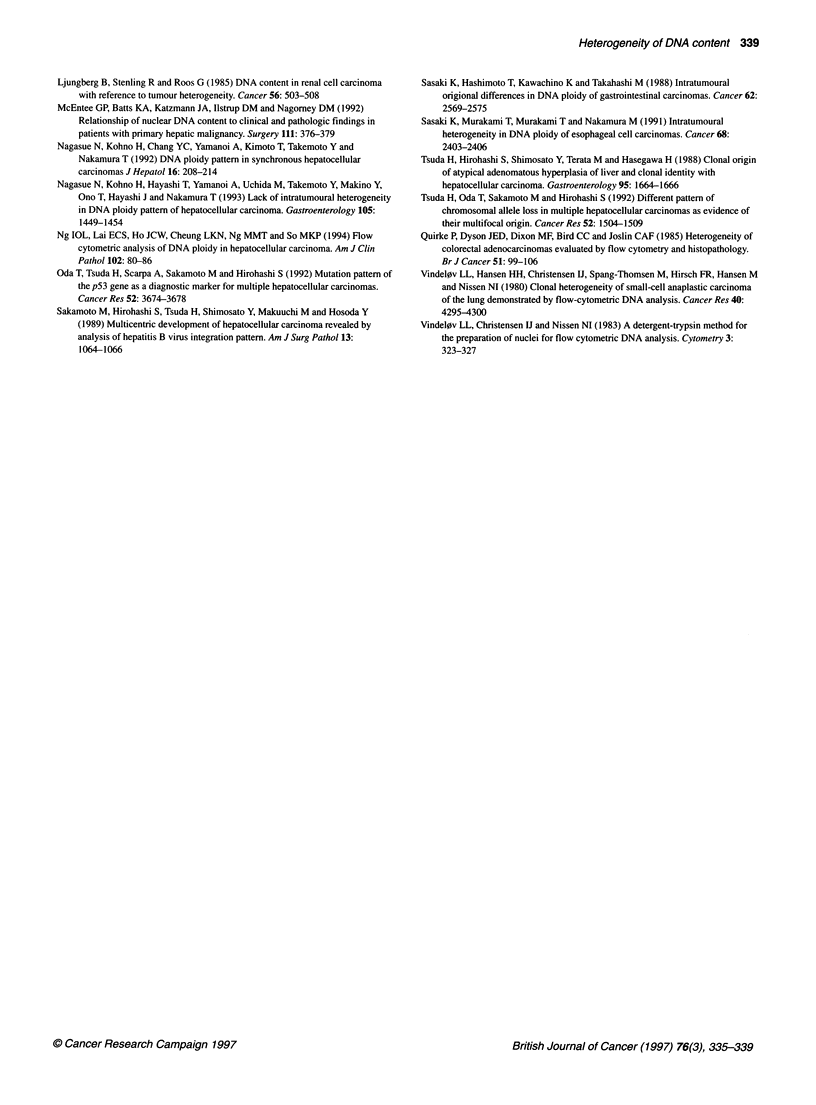


## References

[OCR_00723] Aihara T., Noguchi S., Sasaki Y., Nakano H., Monden M., Imaoka S. (1996). Clonal analysis of precancerous lesion of hepatocellular carcinoma.. Gastroenterology.

[OCR_00728] Chiu J. H., Kao H. L., Wu L. H., Chang H. M., Lui W. Y. (1992). Prediction of relapse or survival after resection in human hepatomas by DNA flow cytometry.. J Clin Invest.

[OCR_00733] Esumi M., Aritaka T., Arii M., Suzuki K., Tanikawa K., Mizuo H., Mima T., Shikata T. (1986). Clonal origin of human hepatoma determined by integration of hepatitis B virus DNA.. Cancer Res.

[OCR_00738] Fujimoto J., Okamoto E., Yamanaka N., Toyosaka A., Mitsunobu M. (1991). Flow cytometric DNA analysis of hepatocellular carcinoma.. Cancer.

[OCR_00741] Hedley D. W., Friedlander M. L., Taylor I. W., Rugg C. A., Musgrove E. A. (1983). Method for analysis of cellular DNA content of paraffin-embedded pathological material using flow cytometry.. J Histochem Cytochem.

[OCR_00748] Hiddemann W., Schumann J., Andreef M., Barlogie B., Herman C. J., Leif R. C., Mayall B. H., Murphy R. F., Sandberg A. A. (1984). Convention on nomenclature for DNA cytometry. Committee on Nomenclature, Society for Analytical Cytology.. Cancer Genet Cytogenet.

[OCR_00754] Hsu H. C., Chiou T. J., Chen J. Y., Lee C. S., Lee P. H., Peng S. Y. (1991). Clonality and clonal evolution of hepatocellular carcinoma with multiple nodules.. Hepatology.

[OCR_00759] Hui A. M., Itabashi M., Kato H., Tachimori Y., Watanabe H., Hirota T. (1994). Flow cytometric DNA analysis of submucosal carcinoma of the esophagus.. Jpn J Clin Oncol.

[OCR_00764] Kanai T., Hirohashi S., Upton M. P., Noguchi M., Kishi K., Makuuchi M., Yamasaki S., Hasegawa H., Takayasu K., Moriyama N. (1987). Pathology of small hepatocellular carcinoma. A proposal for a new gross classification.. Cancer.

[OCR_00770] Kuo S. H., Sheu J. C., Chen D. S., Sung J. L., Lin C. C., Hsu H. C. (1987). DNA clonal heterogeneity of hepatocellular carcinoma demonstrated by Feulgen-DNA analysis.. Liver.

[OCR_00779] Ljungberg B., Stenling R., Roos G. (1985). DNA content in renal cell carcinoma with reference to tumor heterogeneity.. Cancer.

[OCR_00783] McEntee G. P., Batts K. A., Katzmann J. A., Ilstrup D. M., Nagorney D. M. (1992). Relationship of nuclear DNA content to clinical and pathologic findings in patients with primary hepatic malignancy.. Surgery.

[OCR_00788] Nagasue N., Kohno H., Chang Y. C., Yamanoi A., Kimoto T., Takemoto Y., Nakamura T. (1992). DNA ploidy pattern in synchronous and metachronous hepatocellular carcinomas.. J Hepatol.

[OCR_00793] Nagasue N., Kohno H., Hayashi T., Yamanoi A., Uchida M., Takemoto Y., Makino Y., Ono T., Hayashi J., Nakamura T. (1993). Lack of intratumoral heterogeneity in DNA ploidy pattern of hepatocellular carcinoma.. Gastroenterology.

[OCR_00799] Ng I. O., Lai E. C., Ho J. C., Cheung L. K., Ng M. M., So M. K. (1994). Flow cytometric analysis of DNA ploidy in hepatocellular carcinoma.. Am J Clin Pathol.

[OCR_00804] Oda T., Tsuda H., Scarpa A., Sakamoto M., Hirohashi S. (1992). Mutation pattern of the p53 gene as a diagnostic marker for multiple hepatocellular carcinoma.. Cancer Res.

[OCR_00835] Quirke P., Dyson J. E., Dixon M. F., Bird C. C., Joslin C. A. (1985). Heterogeneity of colorectal adenocarcinomas evaluated by flow cytometry and histopathology.. Br J Cancer.

[OCR_00809] Sakamoto M., Hirohashi S., Tsuda H., Shimosato Y., Makuuchi M., Hosoda Y. (1989). Multicentric independent development of hepatocellular carcinoma revealed by analysis of hepatitis B virus integration pattern.. Am J Surg Pathol.

[OCR_00815] Sasaki K., Hashimoto T., Kawachino K., Takahashi M. (1988). Intratumoral regional differences in DNA ploidy of gastrointestinal carcinomas.. Cancer.

[OCR_00820] Sasaki K., Murakami T., Murakami T., Nakamura M. (1991). Intratumoral heterogeneity in DNA ploidy of esophageal squamous cell carcinomas.. Cancer.

[OCR_00825] Tsuda H., Hirohashi S., Shimosato Y., Terada M., Hasegawa H. (1988). Clonal origin of atypical adenomatous hyperplasia of the liver and clonal identity with hepatocellular carcinoma.. Gastroenterology.

[OCR_00830] Tsuda H., Oda T., Sakamoto M., Hirohashi S. (1992). Different pattern of chromosomal allele loss in multiple hepatocellular carcinomas as evidence of their multifocal origin.. Cancer Res.

[OCR_00846] Vindeløv L. L., Christensen I. J., Nissen N. I. (1983). A detergent-trypsin method for the preparation of nuclei for flow cytometric DNA analysis.. Cytometry.

[OCR_00840] Vindeløv L. L., Hansen H. H., Christensen I. J., Spang-Thomsen M., Hirsch F. R., Hansen M., Nissen N. I. (1980). Clonal heterogeneity of small-cell anaplastic carcinoma of the lung demonstrated by flow-cytometric DNA analysis.. Cancer Res.

